# Rapid publication-ready MS-Word tables for two-way ANOVA

**DOI:** 10.1186/s40064-015-0795-z

**Published:** 2015-01-23

**Authors:** Houssein I Assaad, Yongqing Hou, Lan Zhou, Raymond J Carroll, Guoyao Wu

**Affiliations:** Department of Statistics, Texas A & M University, College Station, TX 77843 USA; Department of Animal Science, Texas A & M University, College Station, TX 77843 USA; Hubei Key Laboratory of Animal Nutrition and Feed Science, Wuhan Polytechnic University, Wuhan, 430023 China

**Keywords:** Two-way ANOVA, Multiple comparisons, Online software, Statistical analysis, Biology, Agriculture, R, Shiny

## Abstract

**Background:**

Statistical tables are an essential component of scientific papers and reports in biomedical and agricultural sciences. Measurements in these tables are summarized as mean ± SEM for each treatment group. Results from pairwise-comparison tests are often included using letter displays, in which treatment means that are not significantly different, are followed by a common letter. However, the traditional manual processes for computation and presentation of statistically significant outcomes in MS Word tables using a letter-based algorithm are tedious and prone to errors.

**Results:**

Using the R package ‘Shiny’, we present a web-based program freely available online, at https://houssein-assaad.shinyapps.io/TwoWayANOVA/. No download is required. The program is capable of rapidly generating publication-ready tables containing two-way analysis of variance (ANOVA) results. Additionally, the software can perform multiple comparisons of means using the Duncan, Student-Newman-Keuls, Tukey Kramer, Westfall, and Fisher’s least significant difference (LSD) tests. If the LSD test is selected, multiple methods (e.g., Bonferroni and Holm) are available for adjusting p-values. Significance statements resulting from all pairwise comparisons are included in the table using the popular letter display algorithm. With the application of our software, the procedures of ANOVA can be completed within seconds using a web-browser, preferably Mozilla Firefox or Google Chrome, and a few mouse clicks. To our awareness, none of the currently available commercial (e.g., Stata, SPSS and SAS) or open-source software (e.g., R and Python) can perform such a rapid task without advanced knowledge of the corresponding programming language.

**Conclusions:**

The new and user-friendly program described in this paper should help scientists perform statistical analysis and rapidly generate publication-ready MS-Word tables for two-way ANOVA. Our software is expected to facilitate research in agriculture, biomedicine, and other fields of life sciences.

## Introduction

Since the publication of our paper on rapid generation of .rtf tables from one-way analysis of variance (ANOVA) (Assaad et al. 2014a), we have received multiple requests to construct a similar software package that can handle data arising from two-way ANOVA designs. These statistical tables are common in a variety of scientific applications, including agricultural, biological, and biomedical studies (Steel et al. 1997; Jobgen et al. 2009a; Wang et al. 2014a, b; Meininger and Wu 1997; Wu 1997). An example of such tables is shown in Table 1, reporting the effects of diet [low fat (LF) or high fat (HF)] and body weight classification (lean or overweight) on concentrations of amino acids in the plasma of Sprague-Dawley rats. The table is entirely generated by our program, including the default caption at the bottom. Other table formats are also offered by the software and will be described in details in the subsequent sections (see Table 2, for instance).

**Table 1:** **Effects of diet and weight classification on amino acid concentrations (nmol/ml) in the rat plasma**

**Variable**	**HF**		**LF**		***P*** **-value**		
	**Lean**	**Overweight**	**Lean**	**Overweight**	**Diet**	**Weight**	**D × W** ^**1**^
Asp	45.3 ± 2.39	47.6 ± 2.97	46.2 ± 2.96	45.3 ± 3.45	0.824	0.824	0.604
Glu	88.4 ± 3.23	88.6 ± 1.16	87.8 ± 2.58	87.6 ± 3.18	0.757	0.984	0.951
Asn	128 ± 5.71	114 ± 10.4	120 ± 5.62	133 ± 6.58	0.477	0.922	0.068
Ser	359 ± 10.3^a^	294 ± 4.39^b^	353 ± 7.43^a^	292 ± 3.76^b^	0.576	<0.001	0.807
Gln	562 ± 18.3^b^	645 ± 11.1^a^	559 ± 9.43^b^	655 ± 19.6^a^	0.801	<0.001	0.676
His	124 ± 3.18	115 ± 2.95	120 ± 4.08	130 ± 5.89	0.236	0.906	0.028
Gly	392 ± 6.91^a^	305 ± 7.3^b^	384 ± 7.32^a^	297 ± 5.68^b^	0.254	<0.001	0.955
Thr	379 ± 7.42	359 ± 11.6	381 ± 8.62	376 ± 12.6	0.365	0.223	0.481
Cit	79.9 ± 2.66^a^	53.8 ± 1.63^c^	69.6 ± 3.37^b^	73.9 ± 1.84^ab^	0.057	<0.001	<0.001
Arg	251 ± 8.09^b^	197 ± 6.4^c^	279 ± 7.46^a^	219 ± 4.46^c^	0.001	<0.001	0.642
β-Ala	13.6 ± 0.884^b^	31.4 ± 1.52^a^	13.1 ± 0.611^b^	26.8 ± 1.91^a^	0.064	<0.001	0.123
Taurine	648 ± 17.2^a^	469 ± 13.1^c^	670 ± 11.9^a^	572 ± 12^b^	<0.001	<0.001	0.006
Ala	471 ± 10.2^b^	403 ± 13.6^c^	492 ± 7.54^b^	585 ± 9.22^a^	<0.001	0.252	<0.001
Tyr	131 ± 4.25	135 ± 4.04	125 ± 5.7	139 ± 5.32	0.865	0.069	0.268
Trp	110 ± 3.28	114 ± 4.78	115 ± 2.61	113 ± 3.4	0.658	0.703	0.42
Met	108 ± 5.39	112 ± 3.65	107 ± 4.83	110 ± 4.8	0.77	0.521	0.991
Val	253 ± 9.61^c^	331 ± 6.74^a^	249 ± 10.1^c^	289 ± 9.63^b^	0.017	<0.001	0.048
Phe	111 ± 3.7	108 ± 3.84	106 ± 3.81	113 ± 3.44	0.988	0.645	0.211
Ile	152 ± 4.19^b^	206 ± 4.01^a^	144 ± 4.77^b^	195 ± 3.68^a^	0.035	<0.001	0.674
Leu	212 ± 4.56^b^	308 ± 7.56^a^	216 ± 6.34^b^	302 ± 5.79^a^	0.83	<0.001	0.465
Orn	68.8 ± 1.58^b^	81.4 ± 1.51^a^	67.8 ± 2.06^b^	83.3 ± 1.25^a^	0.786	<0.001	0.381
Pro	277 ± 7.04^b^	342 ± 8.59^a^	286 ± 5.32^b^	334 ± 4.52^a^	0.96	<0.001	0.196
Cys	165 ± 4.53^b^	197 ± 7^a^	157 ± 3.2^b^	194 ± 6.58^a^	0.279	<0.001	0.648
Lys	252 ± 8.82^c^	291 ± 5.17^a^	259 ± 8.32^bc^	282 ± 5.42^ab^	0.957	<0.001	0.273

Values are means ± SEM, n = 9 per treatment group. Male Sprague-Dawley rats (Charles River Laboratories) were fed a low-fat (LF) or high-fat (HF) diet between 4 and 13 weeks of age, as described by Jobgen et al. (2009a, b). At 13 weeks of age, five hours after the last feeding, blood samples were obtained from the tail vein of box-restrained conscious rats using a microhematocrit (Wu 1995). The plasma was analyzed for amino acids using high-performance liquid chromatography (Rezaei et al. 2013; Wu and Meininger 2008). Classification of rats as lean or overweight was performed using the Cluster analysis of body weights, as described by Assaad et al. (2014b).

^a-c^Means in a row without a common superscript letter differ (*P* < 0.05) as analyzed by two-way ANOVA and the TUKEY test. ^1^D × W = Diet × Weight interaction effect.

**Table 2:** **Summary of multiple comparison methods**

LSD	Highest error rate and power of any method. In general, it controls the *FWER in the weak sense*; when there are 3 treatment groups, the FWER is controlled in the strong sense.
DC	Error-rate and power intermediate between SNK and LSD. Controls *the FWER in the weak sense*.
SNK	Error-rate and power intermediate between TK and DC. Controls *the FWER in the weak sense*.
TK	SSP, Lowest error rate and power^*^, controls the FWER in the strong-sense
BF	SSP, Controls the FWER in the strong sense, but it is too conservative (reduces the number of true positives)
Holm	SWP, Stepwise extension of BF; hence, it is more powerful. It should always be preferred over BF; controls the FWER in the strong sense. It doesn’t take logical constraints or correlations into account.
Westfall	More powerful than any MCP controlling the FWER in the strong sense. However, it is computationally expensive.

The table was adapted from Christensen (2011) with modifications.

BF = Bonferroni; DC: Duncan method; LSD = Least significant difference; SNK = Student-Newman-Keuls; TK = Tukey Kramer (or Tukey HSD in balanced designs); SSP = Single-step procedure; SWP = step-wise procedure.

^*^When compared with the classical LSD, SNK, DC.

The present work focuses on generating publication-ready tables from two-way ANOVA models where measurements are summarized as mean ± SEM for each treatment group. Researchers will have an option to include post-hoc test results in these tables using a letter-based algorithm (Piepho 2004) to indicate which treatment groups are significantly different. With this algorithm, treatment means that are not significantly different, as reported by an all-pairwise comparison procedure, are followed by a common superscript letter, e.g., a, b and c (see Table 1). In other terms, two treatments without a common letter are statistically significant at the chosen level of significance α (e.g., α = 0.05 or 0.01). For example, the effects of the HF-Lean and HF-Overweight treatments on the concentration of lysine in the plasma (see last row of Table 1) are significantly different as they do not share a common superscript. On the other hand, LF-Lean and LF-Overweight have the same effect on lysine since they share the superscript ‘b’. By convention, when all treatments have the same effect on a certain response variable, no superscripting is used. This is the case in the first row of Table 1, where all treatments have the same effect on the concentration of aspartic acid (Asp) in the plasma. We believe that our new software will save biologists, and other scientists in general, an ample amount of time by avoiding the manual addition of the superscript letters (see Table 1) derived from the appropriate statistical tests. This offers a distinct advantage over the traditional manual processes for computation and presentation of results in tables that are not only tedious but are also prone to errors.

A Google search of the words “Online two way ANOVA calculator” reveals several online tools^a^ that are capable of performing two-way ANOVA. Despite their user-friendly interface, these programs have serious limitations. Particularly, most of them cannot carry out post-hoc testing of any kind. Some can conduct the Tukey test but do not translate the testing results into a compact letter display; hence the user will have to do the translation by hand. While these online tools may be suitable for pedagogical purposes, their major drawback remains in their inability to export results to an RTF reader in a publication-ready format similar to that of Table 1, making their usage in research impractical and thus unlikely. Also, several software packages (e.g. R, SAS, Stata, SPSS, JMP, etc.) can conduct two-way ANOVA, followed by post-hoc analysis. To our knowledge, none of them is capable of exporting the multiple comparisons results to an RTF reader in a format similar to that of Table 1 without advanced knowledge of the corresponding programming language.

When working on the software, we received considerable and valuable assistance from several R (R Core Team 2014) packages. We would like to acknowledge the formidable efforts on the part of the developers of the following packages: grifExtra (Auguie 2012), XLConnect (Mirai Solutions 2014), agricolae (Mendiburu 2014), rtf (Schaffer 2013), multcomp (Hothorn et al. 2008), plyr (Wickham 2011), ggplot2 (Wickham 2009) and shiny (RStudio 2013). Without the availability of these R packages, this software would not have been developed.

In the remaining sections, we present necessary background materials for two-way ANOVA, followed by a brief summary of multiple comparison techniques. We do not attempt to provide a full description of all testing procedures that have been presented in classical textbooks on experimental designs. Instead, our main goal is to highlight some of the limitations of the statistical tests included in the software and help the researcher decide on a test that is more suitable for his/her data. We would also like to underline the necessary assumptions required by two-way ANOVA and to emphasize that the software should be used only when these assumptions are nearly satisfied. We also illustrate the functionality of the software via a step-by-step approach using different toy datasets to cover most table designs encountered in research papers. The toy datasets are available on the software webpage and can be downloaded from there. Various tips and concluding remarks are given towards the end of this article.

## Background and materials

1. Two-way ANOVA

The main purpose of this section is to present a brief non-technical description of two-way ANOVA and introduce the statistical terms that will be used throughout the rest of this paper. The reader should refer to standard experimental design textbooks for a more in-depth treatment of the subject (Kutner et al. 2005 and Montgomery 2012). Two-way ANOVA, also known as two-factor ANOVA, is concerned with the investigation of the simultaneous effects of two nominal variables, say A and B, called *factors.* These factors can take different values known as *levels.* Each combination of a factor level of A and a factor level of B is a *treatment.* For instance, in Table 1, there are two factors, diet and body weight classification. Factors, diet and body weight, have two levels each, LF and HF for the diet and Lean and Overweight for body weight. This leads to four treatments: LF-Lean, LF-Overweight, HF-Lean, and HF-Overweight. In general, if factor A has *a* levels and factor B has *b* levels, the total number of treatments is *ab*. The variable under study is often referred to as *response* variable. In the same previous example, the amino acids (e.g., Asp, Ser, and Gln), are all response variables. The effect of a factor is defined to be the change in response resulting from a change in the level of the factor. This is frequently called a *main effect.* In some experiments, we may observe that the difference in response between the levels of factor A is not the same at all levels of factor B. When this occurs, there is *interaction* between the two factors. There are three hypothesis of interest in two-way designs, namely, the significance of the main effects of factors A and B, as well as their interaction. The p-values of these tests are reported in the last three columns of Table 1. Two-way ANOVA assume that all observations are independent from each other. Also, measurements corresponding to a treatment group arise from a population having a normal distribution with possibly different means but the same variance across all treatment groups. When interaction is statistically significant (P < 0.05), all pairwise-comparisons are usually carried out on the treatment means. The latter may still be of interest and pairwise-comparisons between treatment means can be made even when the two factors do not interact (Wei et al. 2012). For instance, if diet and weight classification do not interact, it may still be important to determine whether being lean and having a HF intake has the same effect on AA concentrations in the plasma as the LF diet in overweight rats. Our program offers a variety of statistical tests to perform these pairwise comparisons using the cell mean model^b^ (Kutner et al. 2005). *It also gives the option to create the summary table without the post-hoc analysis if it is not of interest to the researcher. In this case, the output will be the same as Table*1*but without the superscript letters*.

2 Multiple comparisons methods

Multiple testing problems arise frequently in biomedical and agricultural research (Hou et al. 2015; Wang et al. 2015a, 2015b), and it is important to address them appropriately. In this section, we merely scratch the surface of the complex topic of multiple hypotheses testing; the interested reader may find the books by Westfall et al. (2011) and Bretz et al. (2010) extremely helpful. These books offer the most up-to-date coverage of the subject and provide a plenty of SAS and R code to help the researcher implement these methods. Hypothesis testing involves two types of errors. A *type I error* (also called *false positive*) occurs when we declare an effect when none exists. Similarly, a *type II error* (*false negative*) occurs if we fail to detect a truly existing effect. *Multiple testing* refers to testing more than one hypothesis in a particular study. Multiple testing procedures are often designed to control the *family-wise error rate* (FWER) of incorrectly rejecting at least one hypothesis in a given group of tests. In other words, the FWER is the probability of committing at least one Type I error in multiple testing. The majority of the classical multiple comparison procedures (MCP), such as DC, LSD and SNK, control the FWER in the *weak sense,* i.e. when the p-values calculations are carried out under the assumption that all null hypotheses are true. In practice, however, it is unlikely that this assumption will hold, therefore allowing the FWER to exceed the usual 5% value. Thus, a stronger control for the FWER under less restrictive assumptions is needed. If, for a given MCP, the FWER is controlled under any partial configuration of true and false null hypotheses, the error is controlled in the *strong sense*. For instance, TK and BF control the FWER in the strong sense but suffer from a low power. Namely, TK and BF are more likely to declare true hypotheses as being true, but might also fail to identify false hypotheses as being false. This trade-off between power and FWER control is the hardest issue to deal with in multiple comparisons. Ideally, it is desired to pick a testing procedure that controls the FWER in the strong sense, while aiming for the highest possible power. Power can be improved by extending *single-step*^*c*^ MCP into *stepwise* procedures via the closure method (Westfall et al. 2011). For example, the stepwise Holm procedure (see step 4 in the next section) is an extension of the single-step BF test. By construction, stepwise procedures are more powerful and control the FWER in the strong sense. MCP with power higher than the Holm procedure are available when there are logical restrictions^d^ among the hypotheses as is the case of all pairwise comparisons. Westfall (1997) extended the Holm’s procedure by incorporating logical restrictions and accounting for random correlations between the hypotheses being tested. Because the method uses extensive simulation to calculate p-values, computation usually requires more time than other MCP. This discussion on multiple comparison methods is summarized in Table 2.

### The software

**Figure 1:**
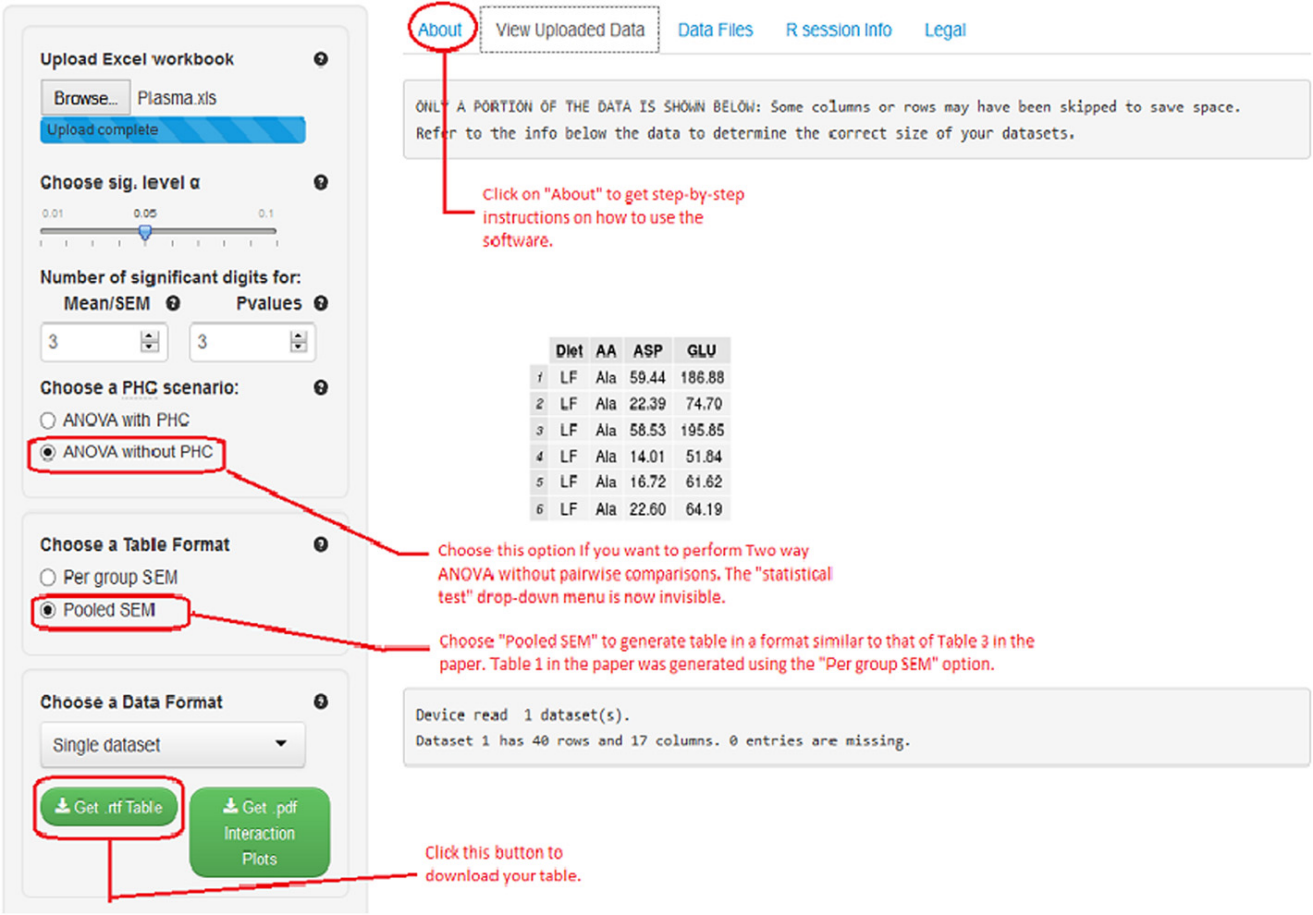
**A screenshot of the software for scenario (S1).** The Excel file Plasma.xls can be downloaded from the “Data Files” panel. Because this file contains a single dataset, the “Single dataset” option is selected (see step 6 above).

**Figure 2:**
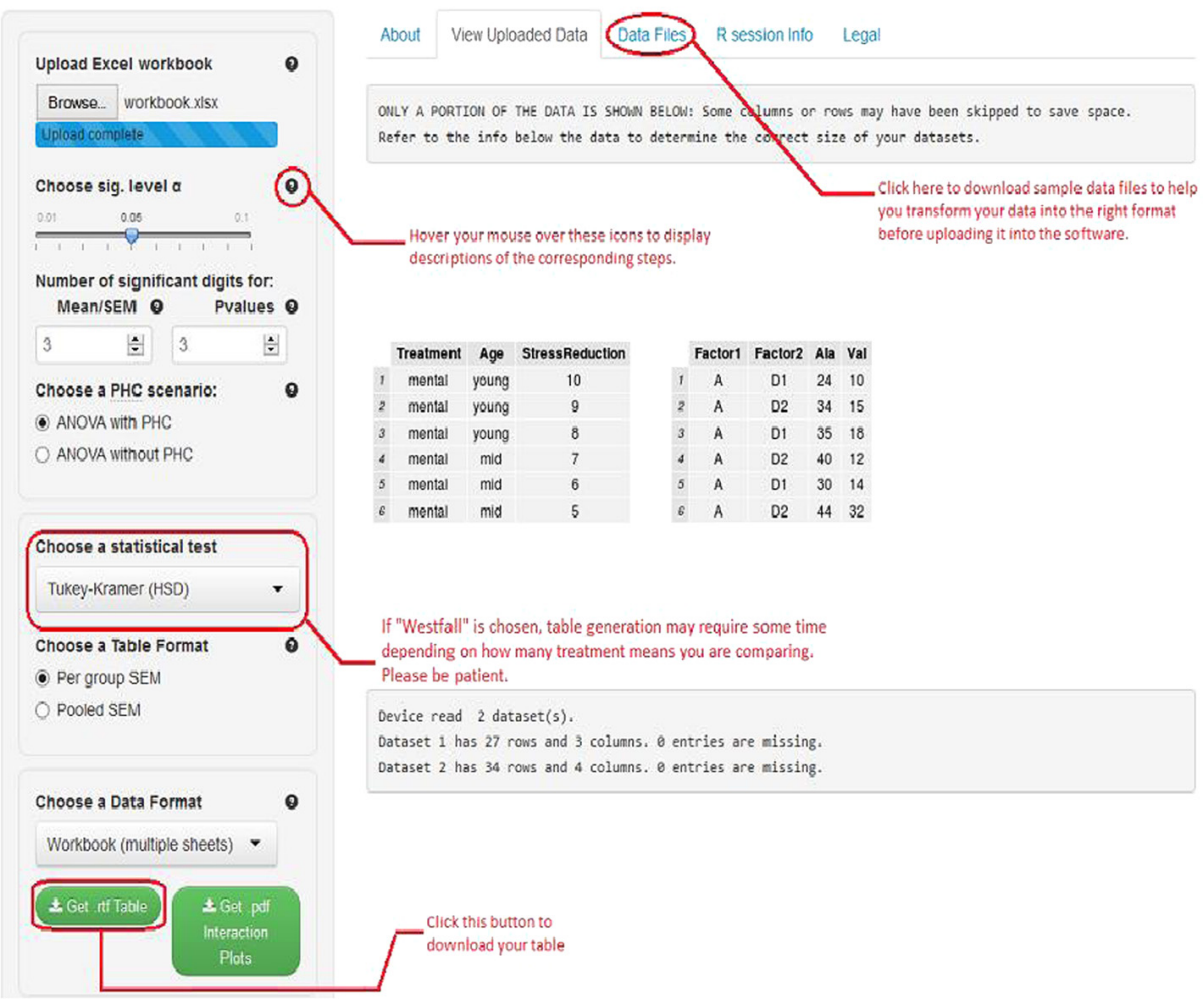
**A screenshot of the software for scenario (S2).** The file workbook.xlsx can be downloaded from the “Data Files” Panel. In the “Choose a Data Format” drop-down menu, the option “Workbook (multiple sheets)” is selected since the file contains multiple sheets/datasets (see step 6 above).

The software (see Figure 1) is available at https://houssein-assaad.shinyapps.io/TwoWayANOVA/. It can handle balanced and unbalanced designs. One complication encountered in unbalanced two-way designs is that p-value computation depends on the order in which factors appear in the dataset. In this case, Everitt and Hothorn (2010) suggest to present two tables corresponding to the different order of appearance of the two factors in the data set. It is worth mentioning that our software program will not work when there is only one observation for each treatment. This is because two-way ANOVA cannot be conducted *unless we assume the two factors do not interact*. Because scientists are interested not only in the main effects of two factors, but also in their interaction, we decided not to include this scenario in our program. The software will, however, display a message alerting the user that two-way ANOVA will not be conducted in this case and a table will not be generated. While the program will work in the presence of *missing values*, it may generate inaccurate SEM values. This is because sample sizes, which appear in the denominator of the SEM, are not the same across different response variables when missing values are present. This issue could be fixed at the expense of sacrificing the sample size display in the caption below each table. As we plan on constantly updating the software, a better solution to handle missing values should be incorporated in the next version when released. Here, we describe, via a step-by-step approach, the detailed functionality of our program. The software can handle two scenarios where data should be arranged accordingly to obtain the correct output. For illustration purposes, data sets corresponding to each scenario can be downloaded from the software webpage under the ‘Data Files’ panel (see Figure 2). We distinguish the following settings:

1. A single dataset in a single-sheet workbook (see file Plasma.xls).
2. Multiple data sets arranged within multiple Excel sheets (one data per sheet) and saved in one Excel workbook (see file workbook.xlsx).

The user of our software should follow the steps below (see Figures 1 and 2):

1. Upload an excel workbook (both .xls and .xlsx format are supported) and select the level of significance α.
2. Indicate the number of significant digits^e^ for the means and SEM and the number of decimal places for the p-values to be displayed in the table. By default, 3 significant digits and 3 decimal places are used for means/SEM and p-values respectively.
3. By default, the software will perform post-hoc pairwise comparisons (PHC) of all treatment means and report the results in each table row. Thus, superscripts are added to each treatment group cell, describing significance or the lack of it. Select “ANOVA without PHC” if you wish to construct a table without PHC in which case the superscripts will not be reported. Note also that, in this case, the “choose a statistical test” drop-down list will disappear as PHC tests will not be conducted.
4. If “ANOVA with PHC” was selected in step 3, specify a statistical test to perform all pairwise comparisons. Currently available tests are Tukey’s HSD, Duncan, Student-Newman-Kleus (SNK), Westfall, and the least significant difference (LSD)^f^. *If you select the (LSD) test, multiple methods, such as Bonferroni (BF) and Holm are available for adjusting p-values. These are actually the Bonferroni and Holm tests described in standard experimental design books*.
5. Choose an output format for the table: Two formats are widely used in the literature. By selecting ‘Per group SEM’, the table will report the mean and SEM for each group (see Table 1). The ‘Pooled SEM’ option will only report the means for each treatment group and one pooled SEM for all treatment groups (see Table 2). The detailed computation of the pooled SEM is provided in the next section.
6. Specify a data format: For (S1), select ‘Single dataset’, whereas for (S2), select ‘Workbook (multiple sheets)’.
7. Click on the ‘Get. rtf table’ to download the table with all statistical results included. *Interaction plots* (only available for the data set in the first sheet in an Excel workbook currently) can also be downloaded in .pdf format by clicking on the ‘Get .pdf interaction plots’ button.

The publication-ready table should now open in the user’s default .rtf reader (e.g. MS Word). The table can now be edited as desired (adding/removing columns, rows or borders, merging, and centering, etc.).

### Regarding the pooled SEM

The main purpose of this section is to describe how the pooled SEM (PSEM) is computed in balanced and unbalanced designs. In general, pooled SEM should only be used when the design is balanced for reasons that will become clear in the definition below. However, we have decided to report a “pooled SEM” for unbalanced designs if the researcher is seeking a more compact table design (one pooled SEM column as opposed to SEM in every treatment column, see Tables 1 and 3). When the design is balanced, i.e. when there are *n* subjects assigned to every treatment, the pooled SEM for any pairwise comparison is the same and is computed as follows:

**Table 3:** **An excerpt from Table** 1 **illustrating the “Pooled SEM” table format**

**Variable**	**HF**		**LF**		**Pooled SEM**	***P*** **-value**		
	**Lean**	**Overweight**	**Lean**	**Overweight**		**Diet**	**Weight**	**D × W** ^**1**^
Asp	45.3	47.6	46.2	45.3	4.2	0.824	0.824	0.604
Ser	359^a^	294^b^	353^a^	292^b^	9.87	0.576	<0.001	0.807
Gln	562^b^	645^a^	559^b^	655^a^	21.6	0.801	<0.001	0.676
Ala	471^b^	403^c^	492^b^	585^a^	14.7	<0.001	0.252	<0.001
Met	108	112	107	110	6.66	0.77	0.521	0.991

Values are means and pooled SEM, n = 9 per treatment group. ^a-c^Means in a row without a common superscript letter differ (*P* < 0.05) as analyzed by two-way ANOVA and the TUKEY test. ^1^D × W = Diet × Weight interaction effect.

$\boldsymbol{PSEM}=\sqrt{\boldsymbol{MSE}\times \frac{2}{\boldsymbol{n}}}$ where MSE is the mean square error. For unbalanced designs, the software will compute a PSEM for each pairwise comparison and then report the highest one. For example, the PSEM for comparing treatment *i* with treatment *j* is calculated using the following formula:

$$ \boldsymbol{P}\boldsymbol{S}\boldsymbol{E}{\boldsymbol{M}}_{\boldsymbol{i}\boldsymbol{j}}=\sqrt{\boldsymbol{M}\boldsymbol{S}\boldsymbol{E}\times \left(\frac{1}{{\boldsymbol{n}}_{\boldsymbol{i}}}+\frac{1}{{\boldsymbol{n}}_{\boldsymbol{j}}}\right)} $$

Where ***n***_*i*_ and ***n***_*i*_ are the sample sizes for treatments *i* and *j*. This process is repeated for every pairwise-comparison and the maximum is reported.

### Common issues and useful tricks

Valid variable or factor level names consist of letters, numbers and the dot or underline characters (typically used to replace spaces between words). All names should start with a letter or the dot not followed by a number. For instance, names such as “.2Ala” are not valid and the software might modify it in order to properly function. Spaces in variable names should be avoided (replace them with a dot or an underscore). *Greek letters should be avoided as they are not rendered properly in the tables. You can add them later on after the table is generated*. Also, if the length of a variable’s name in the dataset is larger than 10 characters, which might be the rule rather than the exception in many cases in biological studies, the software will abbreviate the variable’s name. This can lead to ambiguous or unpleasant terms. We, therefore, advise researchers to subjectively assign descriptive abbreviations for variables with long names before loading their dataset into the software.

#### Factor levels should be described using letters, not numbers

This is how the program distinguishes between numerical variables and categorical/factor variables. For instance, in the sample dataset Plasma.xls, the factor ‘diet’ with the two levels low-fat and high-fat should be coded, for instance as ‘LF’ and ‘HF’, not 0s (for low-fat) and 1s (for high-fat). The latter will lead to an error stating the program was unable to find two factors in your dataset (since one of them is treated as a numerical variable), and thus cannot perform two-way ANOVA.

#### Factors and Levels ordering in the table

By default, the software will use the order in which factors appear in the user’s data set to decide which factor comes on top of the output table. For instance, in Table 1, the factor *diet* with levels *HF* and *LF* occupies the top row, while the factor *weight* with levels *Lean* and *Overweight* comes next. This is because in the data set excel file (Plasma.xlsx), diet appears before weight. Changing the order of appearance in the original data set will be reflected in the output table. Also, the software will use alphabetical order to choose which factor level comes first in the generated table. For example, *HF* appears before *LF* and *Lean* before *Overweight*. If, for some reason, there is a need to change the default level ordering, the following trick is useful: In the data set Excel file, add the number 1 at the beginning of the level to be shown first, the number 2 at the beginning of the level to be shown next, and so on. For example, assume the user wants the level *Overweight* to be shown before *Lean* in Table 1 (thus disobeying the alphabetical order rule). Replace *Overweight* with *1Overweight* and *Lean* with *2Lean* throughout the whole Excel file^g^ (See file Change_Level_order.xlsx). The software will generate a modification of Table 1 shown in Table 4. The user can now delete the added digits.

**Table 4:** **A modification of Table** 1 **that shows how to alter the positions of the levels of the factor “Weight”**

**Variable**	**HF**		**LF**		***P*** **-value**		
	**1Overweight**	**2Lean**	**1Overweight**	**2Lean**	**Diet**	**Weight**	**D × W** ^**1**^
Asp	47.6 ± 2.97	45.3 ± 2.39	45.3 ± 3.45	46.2 ± 2.96	0.824	0.824	0.604
Ser	294 ± 4.39^b^	359 ± 10.3^a^	292 ± 3.76^b^	353 ± 7.43^a^	0.576	<0.001	0.807

Values are means ± SEM, n = 9 per treatment group. ^a-b^Means in a row without a common superscript letter differ (*P* < 0.05) as analyzed by two-way ANOVA and the TUKEY test. ^1^D × W = Diet × Weight interaction effect.

### Concluding remarks

This paper presented a free web-based program capable of generating publication-ready RTF tables for two-way analysis. These tables are often prepared for writing agricultural, biological and medical science papers. Significance statements resulting from an all-pairwise comparison procedure are indicated by the popular superscript letters display, which also allows for the ranking of the treatment means. The software can handle an Excel workbook with multiple datasets saved in multiple sheets, creating one table per dataset. Two of the most commonly used formats for tables (see Tables 1 and 3) in biomedical journals are also supported by our software. The user has full control over the number of significant digits for the treatment means, SEM, and the number of decimal points for the p-values shown in the table. In addition, the program appends an automatic informative caption at the bottom of every table it generates. Due to its user-friendly interface, the software spare researchers a considerable amount of time and eliminate errors introduced by human input. To summarize, this software provide freely available statistics tools to facilitate research in many scientific fields. Future work may involve adding multiple comparisons procedures that are more powerful than Holm but less computationally expensive than Westfall. Also, we may adjust our code to handle two-way ANOVA in the presence of missing data; however, this might be at the expense of not reporting sample sizes in the caption as they are no longer the same across different response variables.

## Availability and requirements

- **Project name:** Rapid publication-ready MS Word tables for two-way ANOVA.
- **Project home page:**https://houssein-assaad.shinyapps.io/TwoWayANOVA/
- **Operating system(s):** Platform independent.
- **Programming language:** R, HTML/CSS, RTF.
- **Other requirements:** internet connection, Mozilla Firefox, or Google Chrome.
- **Any restriction to use by non-academics:** None.

## Endnotes

^a^See http://faculty.vassar.edu/lowry/anova2x2.html, http://scistatcalc.blogspot.com/2013/11/two-factor-anova-test-calculator.html.

^b^Assume there are two factors A and B with *a* and *b* levels, respectively. In the cell mean model, the two factors are collapsed into one factor with *ab* levels (each level correspond to a treatment combination) and computation is now carried out using One-way ANOVA.

^c^When testing multiple hypotheses, a test procedure is called a *single-step* method if the rejection or non-rejection of a null hypothesis does not take the decision of any other hypothesis into account, e.g. the BF and TK tests. On the other hand*, step-wise* methods differ from single-step procedures in that the results of a given test depend upon the results of other tests, e.g., Holm.

^d^For instance, in a three group ANOVA, H_12_: M_1_ = M_2_, H_13_: M_1_ = M_3,_ and H_23_: M_2_ ≠ M_3_ cannot be simultaneously true. Thus the hypotheses are *logically restricted*. Choosing a test that does not account for these *logical constraints* might lead to loss in power and problems with the interpretation of the test results.

^e^For the sake of completeness, we summarize the basic rules for significant digits.

1. All nonzero digits are significant.
2. All zeros between significant digits are significant.
3. All zeros which are both to the right of the decimal point and to the right of all non-zero significant digits are themselves significant.

For example, 3211 has four significant digit (using rule 1), 400.06 has five significant digits (using rules 1 and 2), 3.2000 has five significant digits (using rules 1 and 3), and 0.003 has 1 significant (using rule 1).

^f^The LSD test is carried out by choosing the LSD option in step 4 and setting the method for adjusting p-values to ‘none’ (which is the default selection).

^g^Use the “Find and Replace” feature in Excel to achieve this task. This feature can be accessed on PC by clicking CTRL + F and then go to the “Replace” tab.

## Abbreviations

ANOVA: Analysis of variance
SAS: Statistical analysis system
SD: Standard deviation
SEM: Standard error of the mean
PSEM: Pooled standard error of the mean
AA: Amino acids
LF: Low-fat
HF: High- fat
